# Satellite cloud image segmentation based on lightweight convolutional neural network

**DOI:** 10.1371/journal.pone.0280408

**Published:** 2023-02-06

**Authors:** Xi Li, Shilan Chen, Jin Wu, Jun Li, Ting Wang, Junquan Tang, Tongyi Hu, Wenzhu Wu

**Affiliations:** 1 Foundation Department, Chongqing Medical and Pharmaceutical College, Chongqing, China; 2 School of Communication and Information Engineering, Chongqing University of Posts and Telecommunications, Chongqing, Chongqing, China; 3 Chongqing Botong Water Conservancy Information Network Co.,Ltd., Chongqing, China; Menoufia University, EGYPT

## Abstract

More than 50% of the images captured by optical satellites are covered by clouds, which reduces the available information in the images and seriously affects the subsequent applications of satellite images. Therefore, the identification and segmentation of cloud regions come to be one of the most important problems in current satellite image processing. Due to the complexity and variability of satellite images, especially when the ground is covered with snow, the boundary information of cloud regions is difficult to be accurately identified. The fast and accurate segmentation of cloud regions is a difficult point in the current research. We propose a lightweight convolutional neural network. Firstly, channel attention is used to optimize the effective information in the feature maps as a way to improve the network’s ability to extract semantic information at each scale. Then, we fuse high and low-dimensional feature maps to enhance the network’s ability to obtain small-scale semantic information. In addition, the feature aggregation module automatically adjusts the input multi-level feature weights to highlight the details of different features. Finally, we design the fully connected conditional random field to solve the problem that some noise in the input image and local minima during training is passed to the output layer resulting in the loss of edge features. Experimental results show that the proposed method achieves 0.9695 and 0.8218 for overall accuracy and recall, respectively, which has higher segmentation accuracy with the shortest time consumption compared with other state-of-the-art methods.

## 1. Introduction

Ground objects in satellite images can be obscured by clouds, which affects the imaging quality of satellite images and causes difficulties for us to obtain the information about ground objects under the clouds [1–3]. It brings greater impact in the subsequent applications of satellite images such as object tracking and change detection [4–7]. In order to obtain more useful information in satellite images, the identification of cloud regions is one of the urgent problems in the current satellite image processing. In recent years, researchers have developed many cloud detection methods. Ref [8] proposed a method that combines spectral reflectance with background information, which compares the detection results with a generated reference cloud mask for pixel-level verification. This method has good results in cloud image recognition, but it needs to improve the computational efficiency and accuracy by comparing the effectiveness of different texture features. Ref [9] used a dual projection approach to predict the cloud shadow shape on the slope side and applies terrain correction to remove terrain shadows and estimate the cloud base height of neighboring clouds. Both of them will reduce the possibility of cloud and cloud shadow mismatch and improve the accuracy of cloud shadow detection in places with large terrain gradients. This method is sensitive to temperature and altitude, and tends to confuse cloud areas with ground shadows, Meanwhile, it has poor detection accuracy in mountainous areas. Ref [10] fused spectral, texture, and structural information and learns depth discriminative features from a large amount of selected information, and then uses a fuzzy function to map the learned features to the corresponding cloud density maps. Although this method obtains higher cloud detection accuracy for different spatial resolutions and various ground surfaces, texture and structure features are manually selected. It may not contain enough information for poor detection accuracy in some specific cases. Ref [11] proposed a for k-means classification method to determine cloud pixels by taking the pixel with the nearest mean value of each class as the initial clustering center of the k-means algorithm and determining the class of the clustering result based on the class of the initial clustering center. This method reduces the error caused by the random selection of initial clustering centers, but the k-means algorithm is very sensitive to the selection of initial clustering centroids, and the initial classification objects still need to be selected manually in many cases.

In general, the traditional image segmentation methods require manual design and feature extraction, which contain only part of the image information, and have less versatile. In recent years, deep learning methods have been widely used for image segmentation, among which full convolutional networks [12] have made significant progress in image segmentation. The full convolutional neural network achieves pixel-level classification of images, thus it solves the problem of semantic-level image segmentation. It can accept an input image of arbitrary size and upsample the last feature map using a convolutional layer to recover it to the same size as the input image so that predictions can be generated for each pixel. In addition, it preserves the spatial information in the original input image and classifies the pixels in the upsampled feature map, and its image segmentation accuracy is much higher than that of traditional segmentation algorithms.

Ref [13] segmented the original image with superpixels and used a convolutional neural network (CNN) to extract multiscale features from each superpixel. This method achieves predictive classification of all superpixels in an image by optimizing the initial clustering centers and expanding the search space to obtain accurate cloud boundaries. Ref [14] proposed a depth pyramid network based structure to solve the problem of clouds without distinct spectral features in RGB color images by using texture information of image pixels in cloud and non-cloud regions. Ref [15] designed a super-pixel-level cloud detection method with CNN and deep forest, which makes full use of low-level features such as color and texture information to classify each pixel in a remotely sensed image. This method obtained good results in cloud region detection task, but the generalization ability of the model is weak. Ref [16] proposed a full convolutional neural network method for gradient-based recognition to accomplish the identification and separation of cloud-snow regions, using the weights of the trained network to detect cloud pixels in an end-to-end manner. This method is weak for multi-scale feature extraction of images and the segmentation accuracy needs to be improved. Ref [17] proposed a CNN model with a symmetric encoder-decoder structure. The encoder network combines low-level cloud features to form high-level, low-resolution cloud feature maps, whereas the decoder network restores the obtained high-level cloud feature maps to the same resolution of input images. This method has high accuracy for segmentation of cloud images, but the recognition accuracy of thin cloud layers is poor.

The field of view of satellite images at equal resolution is much larger than that of ground acquired images, resulting in more small-scale targets in aerial images. So the semantic segmentation network applied to satellite images should have stronger ability to extract small-scale semantic information [18–20]. Aerial remote sensing applications have real-time requirements for image semantic segmentation algorithms, such as aerial search and rescue, which requires algorithms to quickly identify people who need to be rescued to avoid delaying the best time for rescue, but aerial images have several times the resolution of ground-captured images, so the network used to process aerial images must have a faster operation speed. The network based on pyramid structure runs slowly and the multi-branch network segmentation accuracy is not high [21–23]. There are no excessive irregular boundaries between neighboring targets in satellite cloud images and less detailed information in the images, which makes the spatial information of both high and low dimensional feature maps more accurate. Therefore, we will not introduce the spatial attention branch to optimize the spatial information of low-dimensional feature maps, nor the pooling fusion module to eliminate the spatial information gap between high and low-dimensional feature maps, and only use the channel attention branch to optimize the semantic information of each dimensional feature map [24, 25]. Hammad et al. fused CNN and SVM classifiers to enhance the feature extraction ability of the model and improved the activation function to enhance the convergence speed of the model [26, 27]. Elgendy et al. designed Q-learning and Deep-Q-Network algorithms to optimize the computational cost of the model [28–30].

For feature fusion, shallow features contain more local information such as color, texture and boundary are richer. While deep features have lower resolution, they have rich semantic information. Direct fusion of features at different levels leads to retaining more local information in the newly fused features, which can easily be regarded as background noise and affect the accuracy of target region recognition. Ref [31] fuses multi-level features directly to improve the accuracy of the network in recognizing target regions. Ref [32] uses fewer convolutional layers in feature fusion to handle high-resolution network branches. Ref [33] integrates different features into multiple resolutions as a way to achieve target region recognition at a specific resolution. Ref [34] introduces a pyramid pooling module to fuse multi-level features using pooling operations of different sizes. However, the above-mentioned feature aggregation methods do not assign the weight values of different features according to their importance, and feature fusion has little improvement on network performance.

**Fig 1:**
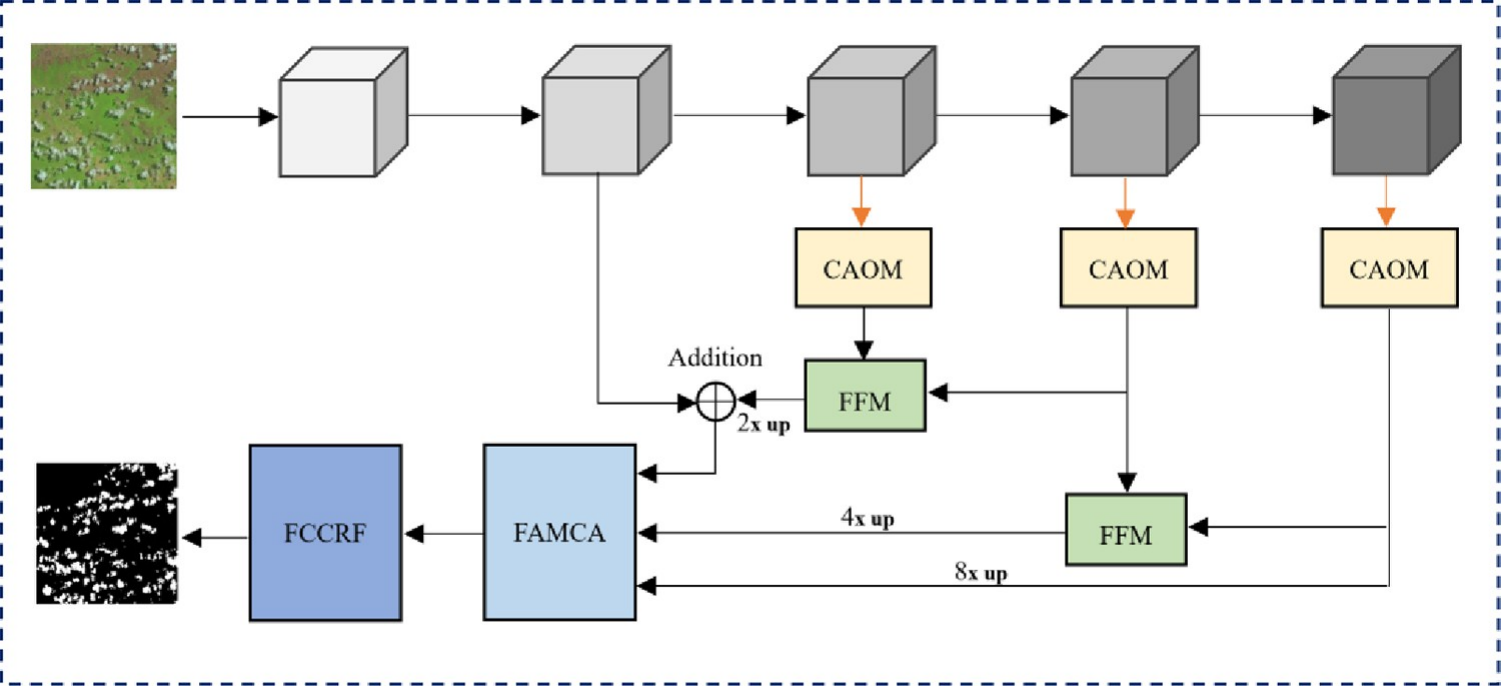
The main framework of our proposed network.

To solve the above problems, we propose a network based on attention mechanism and multi-feature fusion, and the overall structure is shown in Fig 1. Firstly, the backbone network is used to encode the semantic information of the satellite images, and the channel attention optimization module (CAOM) is used to optimize the feature maps at different levels in the baseline network to improve their ability to capture the semantic information. Secondly, the output different feature maps are fed into the feature aggregation module (FFM), which significantly improve the ability of low-dimensional feature maps to capture small-scale semantic information. In addition, the feature aggregation module with channel attention (FAMCA) module gives different weights to each input feature in the process of fusing different features, which enables the network to spontaneously perceive multi-scale features. Finally, we design the fully connected conditional random field to solve the problem that some noise in the input image and local minima in the training process can be passed to the final output layer by the network, resulting in the loss of edge features.

## 2. Data and methods

### 2.1. Dataset

**Fig 2:**
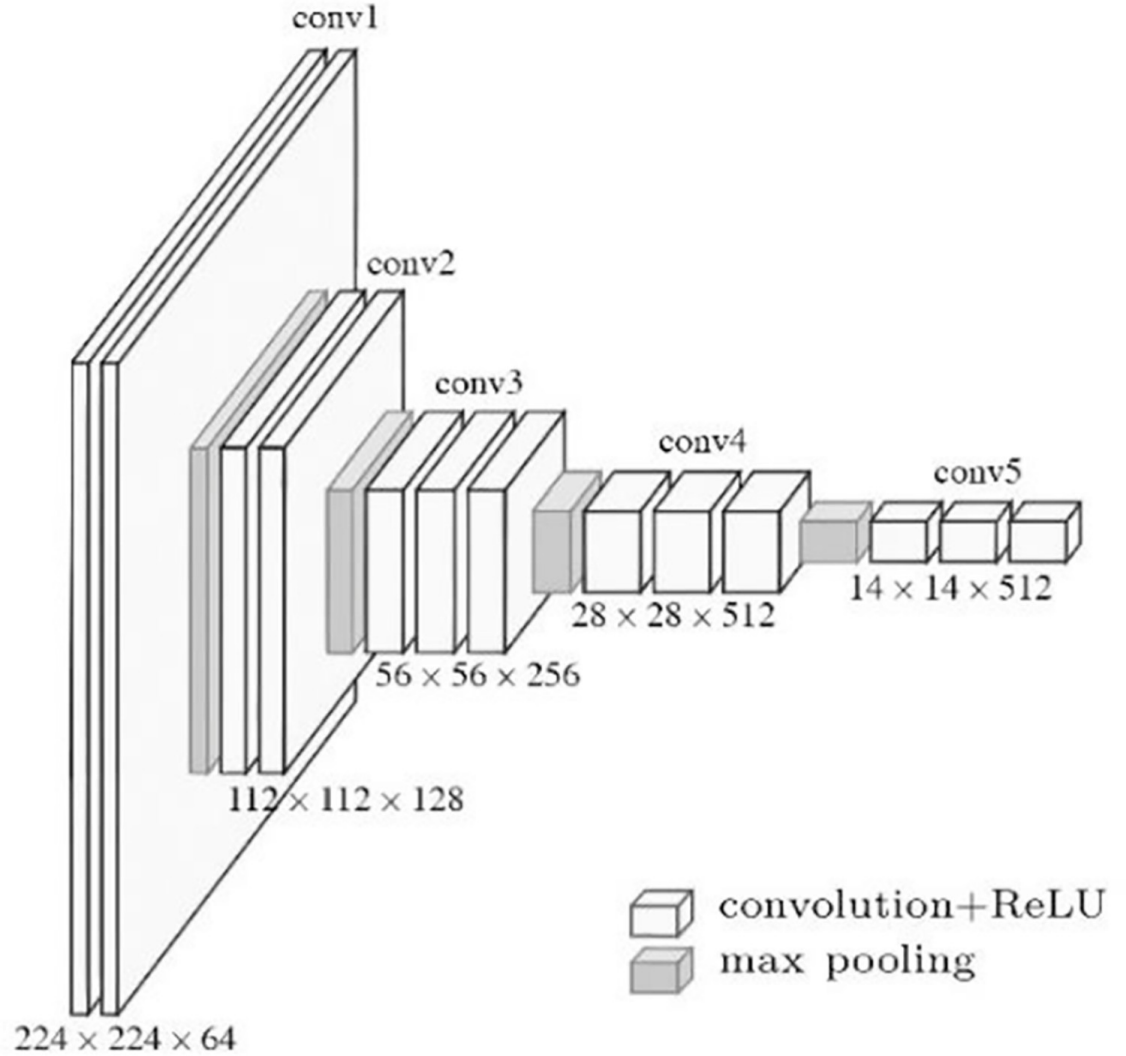
Structure diagram of VGG-16.

We used the 38-Cloud dataset for training and testing, which extracted 23 satellite images (each approximately 185 km×185 km) from 75 Landsat8 Collection 1 in North America, as shown in Fig 2, of which 18 images were used for training and 5 images were used for testing. Among these 23 images, each image of the dataset was cropped into 384 × 384 blocks to obtain 8400 blocks for training and 2300 blocks for testing. The annotation of this dataset was done by the School of Engineering Science, Simon Fraser University, Burnaby, BC, Canada.

### 2.2. Methods

The CNN-based segmentation method that does not require assumptions and prior knowledge can automatically learn features of the target, and thus it performs significantly better than traditional methods. In this section, we describe the channel attention optimization module, the feature fusion module, and the feature aggregation module with channel attention.

**Fig 3:**
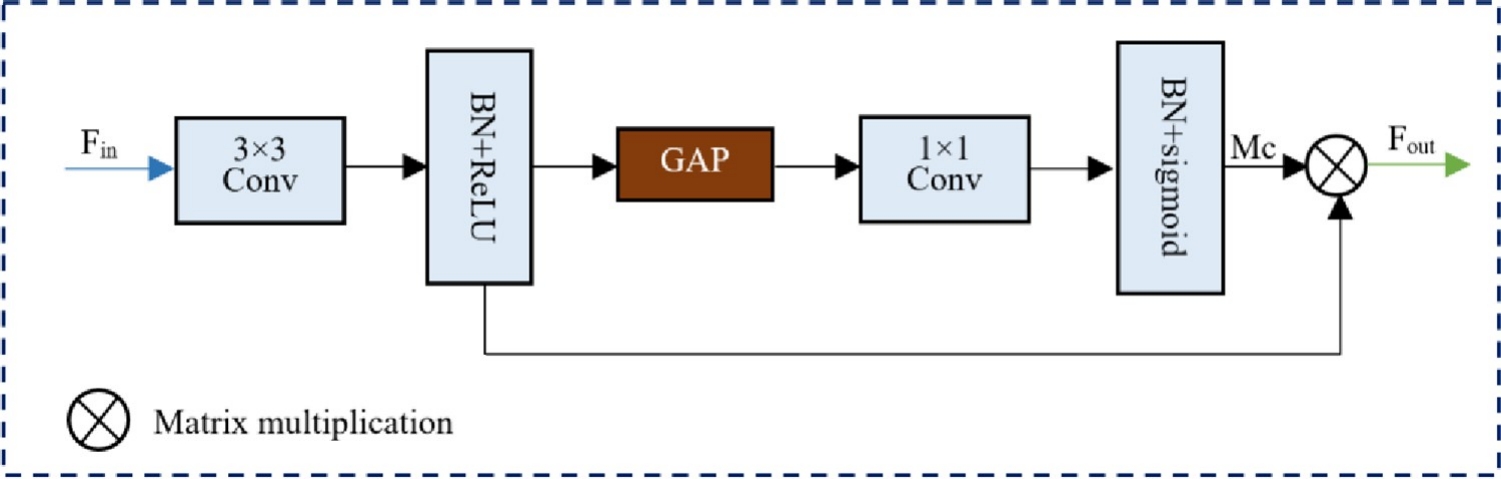
The channel attention optimization module (CAOM).

We use VGG-16 as the backbone, and the results are shown in Fig 2. Since the network depths of VGG-16 are all shallow and have limited ability to capture semantic information, directly fusing its intermediate feature maps to obtain multi-scale semantic information will lead to poor segmentation results, especially for small-scale targets. Therefore, before fusing multi-level feature maps, each dimensional feature map is optimized using CAM, which first feeds the feature map F_in_ into a 3 × 3 convolutional process with BN and ReLU to unify the number of output channels of different dimensional feature maps. Then, the global average pooling (GAP) is used to reduce the resolution of the feature map to 1×1, and the 1×1 convolutional layer combining BN and Sigmoid is used to process the output of GPA to generate the channel attention mask Mc. Finally, Mc is multiplied with the input feature map to generate the output of the channel attention branch, and the overall process is shown in Fig 3.

**Fig 4:**
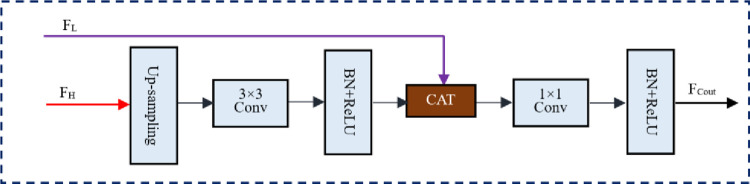
The feature fusion module(FFM).

Although the channel attention optimization branch can be used to improve the information capture capability of the feature maps, the improvement is limited and the network is still unable to extract accurate small-scale semantic information. After optimizing the feature maps in each dimension using CAOM, FFM is continued to be used to optimize the shallow feature maps. FFM enhances the ability of the shallow feature map to obtain abstract information by introducing information from the deep feature map into the shallow feature map, which allows the shallow feature map to obtain more accurate small-scale semantic information. The overall structure of FFM is shown in Fig 4. We use a fusion module with two input feature maps F_L_ and F_H_, where the resolution of the deep feature map F_H_ is 1/2 the resolution of the shallow feature map F_L_, which can limit the information gap between the two feature maps and facilitate information fusion. First, the fusion module upsamples the resolution of F_H_ to be consistent with the shallow feature map by upsampling. Then, the 3×3 convolutional layer combining BN and ReLU is used to optimize the deep feature map after upsampling, and the optimized deep feature map and deep feature map are aggregated by CAT. Finally, the 1×1 convolutional layer combining BN and ReLU is used to optimize the aggregated results, and the output is F_cout_. With the fusion module, the shallow feature map can obtain more abstract information, and its semantic information capturing ability will be significantly improved. In addition, the fusion module is simple in structure and small in computation, which can quickly improve the ability of shallow feature maps to capture small-scale semantic information.

**Fig 5:**
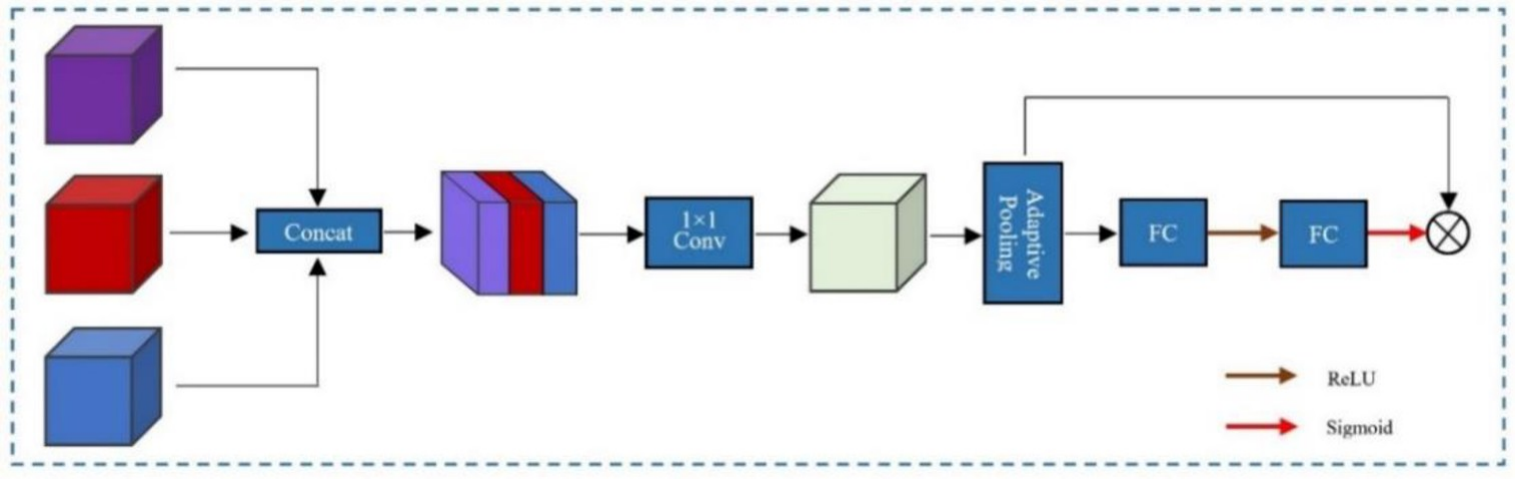
The feature aggregation module with channel attention(FAMCA).

The input of the channel attention-based feature aggregation module three different dimensions of features. After obtaining multi-scale features, not all of them can improve the network performance. We need to increase the weights of important features in feature aggregation while suppressing useless information, and simple matrix addition and multiplication cannot achieve the above functions. Therefore, we propose an aggregation module that automatically adjusts the three input weights using an attention mechanism, as shown in Fig 5.

Firstly, we perform the join operation on three feature maps X_1_∈R^C×H×W^, X_2_∈R^C×H×W^ and X_3_∈R^C×H×W^ to obtain the fusion feature called X_C_∈R^3C×H×W^. Then, we convolve *X*_*C*_ using a convolution kernel of size 1 × 1 to obtain X_C1_∈R^C×H×W^. Finally, we use the adaptive averaging pooling layer to generate channel statistics to adaptively enhance informative features and suppress useless features, and the statistics are denoted as S_F_∈R^C×1×1^, as shown in Eq 1.


$$S_{F}=\frac{1}{H\times W}\sum_{i=l}^{H}\sum_{j=l}^{W}X_{C1}(i,j)$$ (1)

We use two fully connected layers and two activation functions to capture the weights between the feature mappings, as shown in Eq 2.


$$A_{C}={\sigma (f}_{c2}(\varphi {(f}_{c1}(S_{F}))))$$ (2)

Where A_C_∈R^C×1×1^ is the expected range of attention for [0,1], and *φ* and *σ* are the ReLU and sigmoid activation functions, respectively. Then we generate the final feature mapping by element-level multiplication.


$$X_{F}=S_{F}∙A_{C}$$ (3)

### 2.3. Fully connected conditional random field

The probability map of the network output becomes smooth due to the increase of the perceptual field and the learned spatial context. Some of the noise in the input image and the local minima that appear in the training process are passed to the final output layer by the network, which will lead to the loss of edge features and thus affect the segmentation effect. To solve the above problem, we design a fully connected conditional random field (FCCRF) to transform the image segmentation problem into an optimization problem and use the minimization energy function to solve it. The set of pixels of the input image is denoted as I_*i*_, and its label assignment function is Y. The energy function can be expressed as

$$E(Y)=\sum_{i=1}^{M}\emptyset (y_{i}^{u})+\sum_{\vee i,j,i\neq j}^{M}\Psi (y_{i}^{u},y_{i}^{v})$$ (4)

Where $y_{i}^{u}$ is the probability value of assigning label u to pixel I_*i*_ and $y_{i}^{v}$ is the probability value of assigning label v to pixel I_j_. The one-dimensional potential function $\emptyset (y_{i}^{u})$ is a negative log-likelihood function that measures the cost of assigning label u to pixel I_*i*_ and can be expressed as

$$\emptyset (y_{i}^{u})=‐\mathrm{logP}(u|I_{i})$$ (5)

Where *P*(*u|*I_*i*_) denotes the probability value of pixel I_*i*_ belonging to label u, which is output by the convolutional neural network. The binary potential function $\Psi (y_{i}^{u},y_{i}^{v})$ uses the form of a fully connected graph to measure the cost of assigning labels u,v to pixels I_i_, I_j_ simultaneously, which can be expressed as

$$\Psi (y_{i}^{u},y_{i}^{v})=\gamma (u,v)k(f_{i},f_{j})$$ (6)

By defining the cost function as a linear combination of a set of Gaussian kernels, the model can be expressed as

$$\Psi (y_{i}^{u},y_{i}^{v})=\gamma (\mathrm{u,v})\sum_{m=1}^{K}w^{m}k^{m}(f_{i},f_{j})$$ (7)

Where γ(u,v) denotes the compatibility of labels u and *γ*(*u*, *v*) = 1 when u≠v. *w*^*m*^ denotes the weight of Gaussian kernel function k^m^ and f_i_, f_j_ denotes the feature vector of pixel pair I_i_, I_j_. In the multiclass segmentation task using a dual kernel potential function i.e. K = 2, where the appearance Gaussian kernel can be expressed as

$$k^{1}=exp(-\frac{{{|p}_{i}-p_{j}|}^{2}}{2\theta_{\alpha }^{2}}-\frac{{{|e}_{i}-e_{j}|}^{2}}{2\theta_{\beta }^{2}})$$ (8)

The smoothness Gaussian kernel is expressed as

$$k^{2}=exp(-\frac{{{|p}_{i}-p_{j}|}^{2}}{2\theta_{\gamma }^{2}})$$ (9)

Where *p*_*i*_, *p*_*j*_ denote the spatial coordinates of pixels I_i_, I_j_ respectively, *e*_*i*_, *e*_*j*_ denote the intensity of pixels I_i_, I_j_ respectively.

### 2.4. Loss function

We use the binary cross-entropy loss function.


$$L=‐[\mathrm{ylog}\hat{y}+(1‐y)\log\left(1‐\hat{y}\right)]$$ (10)

Where $\hat{y}$ is the predicted value and y is the ground-truth.

### 2.5. Evaluation metrics

We implement the proposed network based on the PyTorch with an RTX A4000 GPU. We adopt VGG-16 as our backbone network. The proposed network is trained using the Adam optimizer with an initial learning rate of 10^−4^, and is trained for 50 epochs in total.

To effectively evaluate the performance of our model, we use the Jaccard index, precision, recall, Overall Accuracy (OA), Dice and Hausdorff distance (HD)for validation. These indices are defined in detail as follows:

$$\mathrm{Jaccard}\;\mathrm{Index}=\frac{\mathrm{TP}}{\mathrm{TP}+\mathrm{FN}+\mathrm{FP}}$$ (11)

$$\mathrm{Precision}=\frac{\mathrm{TP}}{\mathrm{TP}+\mathrm{FP}}$$ (12)

$$\mathrm{Recall}=\frac{\mathrm{TP}}{\mathrm{TP}+\mathrm{FN}}$$ (13)

$$\mathrm{OA}=\frac{\mathrm{TP}+\mathrm{TN}}{\mathrm{TP}+\mathrm{TN}+\mathrm{FP}+\mathrm{FN}}$$ (14)

$$\mathrm{Dice}=\frac{2\mathrm{TP}}{\mathrm{FP}+2\mathrm{TP}+\mathrm{FN}}$$ (15)

Where TP, TN, FP and FN are the total number of true positive, true negative, false positive and false negative pixels, respectively.

The Hausdorff distance (HD) can be defined as follows.


$$\mathrm{HD}=\max[d_{\mathrm{AB}},d_{\mathrm{BA}}]$$ (16)

$$d_{\mathrm{AB}}=\max_{a\in A}\;\min_{b\in B}\|a-b\|$$ (17)

$$d_{\mathrm{BA}}=\max_{b\in B}\;\min_{a\in A}\|b-a\|$$ (18)

Where A denotes the real result image, B denotes the predicted result image, and *d*_*AB*_ and *d*_*BA*_ denote the one-way Hausdorff distance between the real segmented image and the predicted segmented image, respectively. HD is the maximum value in *d*_*AB*_ and *d*_*BA*_, which is the maximum mismatch degree between the predicted segmented image and the real segmented image, and the smaller the value indicates that the network segmented image is closer to the real labeled image.

## 3. Experimental results

**Fig 6:**
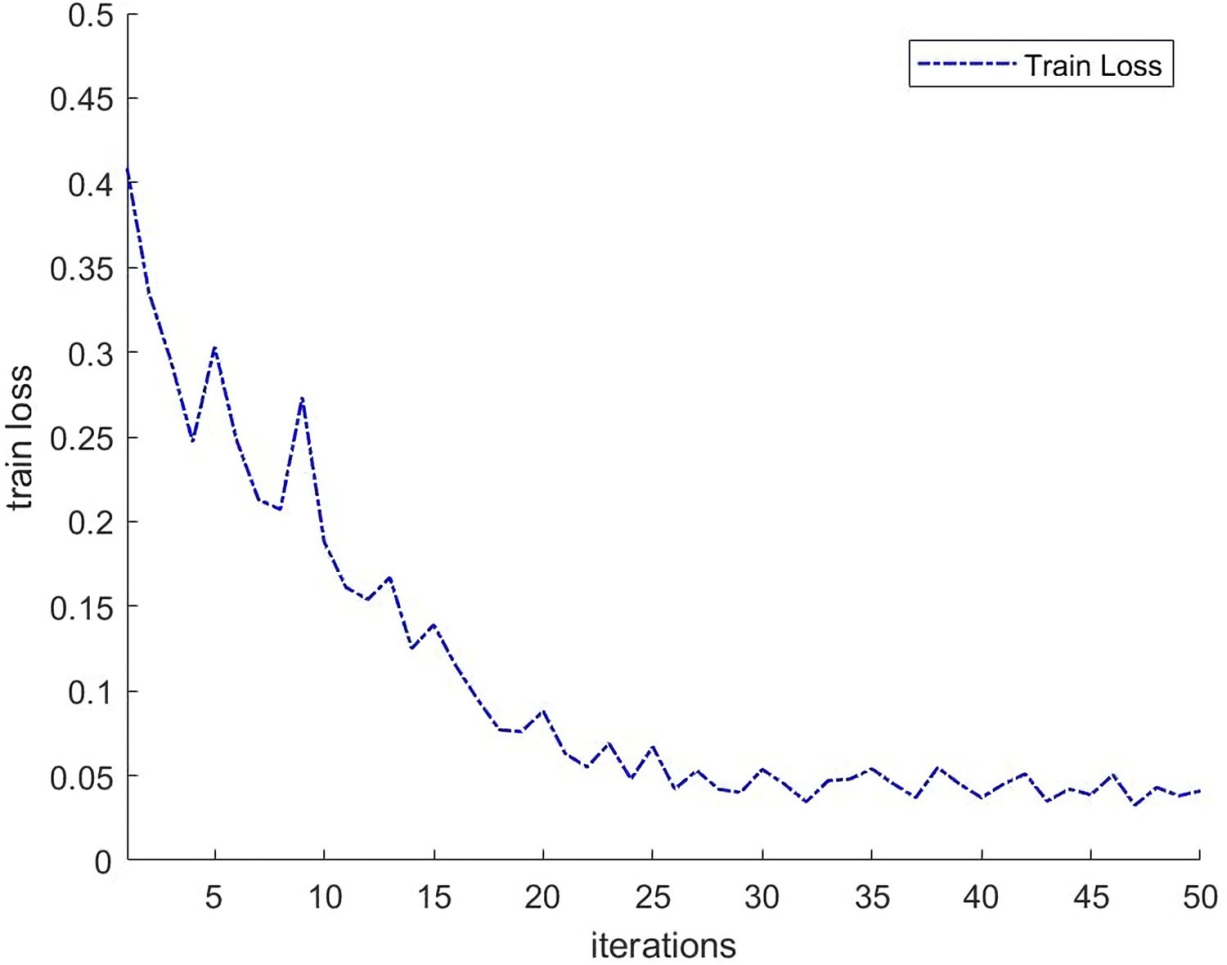
Train loss.

**Fig 7:**
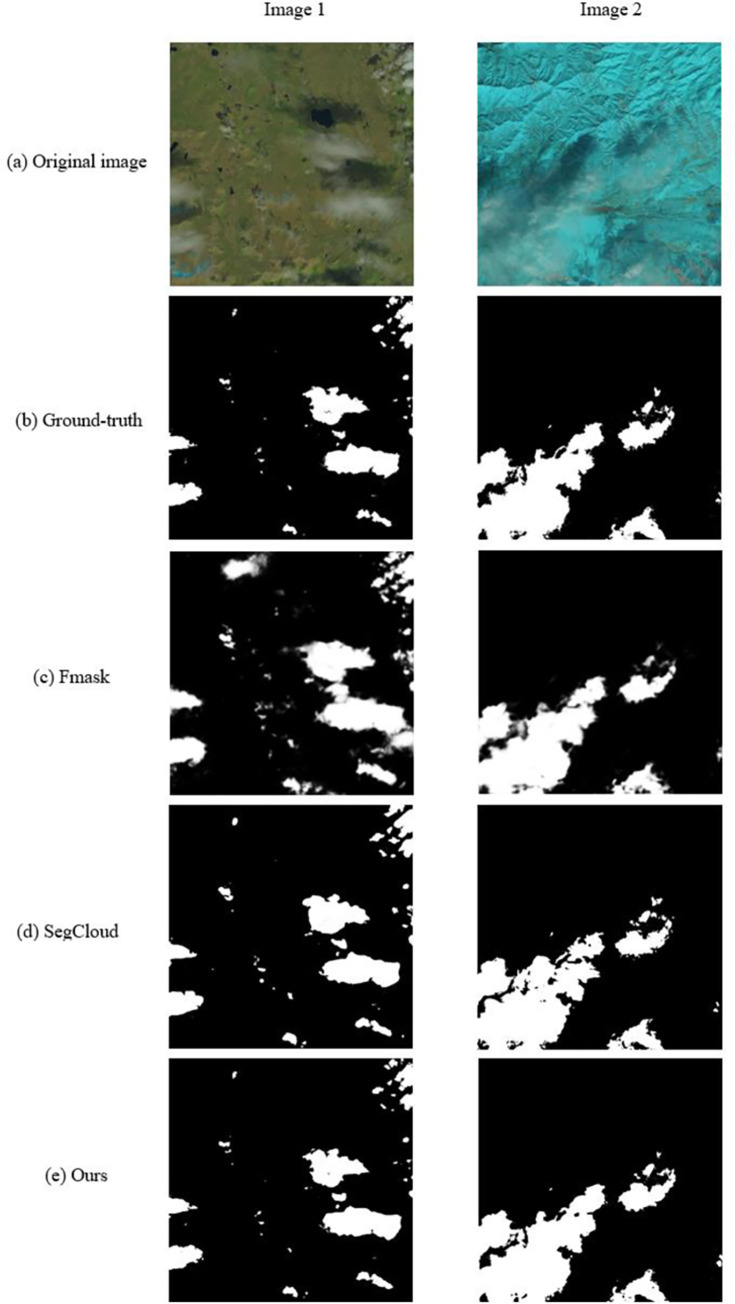
Visual comparison of state-of-the-art satellite cloud image segmentation methods. From top to bottom, (a) are 2 different satellite cloud images, (b) are the ground-truth corresponding to satellite cloud images, (c) are the results of Fmask [9], (d) are the results of SegCloud [17], (e) are the results of our method.

In this section, we compare the proposed network with existing satellite cloud segmentation methods as a way to demonstrate the effectiveness of the method in this paper. The network training loss function is shown in Fig 6. The proposed method in this paper is compared with two state-of-the-art satellite cloud image segmentation methods, Fmask [9] and SegCloud [17], and their segmentation results are shown in Fig 7. It can be seen that the method proposed in this paper can segment the cloud regions in satellite images at different scales more accurately.

Since the edges of the cloud region on the right side of image 1 are blurred, FMask [9] fails to segment the blurred boundary correctly, and SegCloud [17] segmentation results are relatively good, so it can be seen that the traditional method is difficult to identify the edges of the cloud region accurately. When the ground surface is covered by snow and ice, the contrast between cloud and snow region images is low, and both FMask and SegCloud are prone to incorrectly identify snow and ice regions as cloud regions. For isolated small-sized cloud regions, FMask and SegCloud have large segmentation errors. The network proposed in this paper enhances the obtained multiscale features and the association information between them, and automatically adjusts the input weights of multidimensional features by the FAMCA module. The network can effectively highlight the details of different features, which makes the recognition of cloud image regions more accurate.

The Jaccard index, Precision, Recall, OA and time cost (TC) of processing images using different methods are shown in Table 1. As it can be seen, the proposed method in this paper has the highest image segmentation accuracy with least time consumption. In other words, our network can better distinguish the target and background with guaranteed segmentation speed. This also shows that CAOM and FFM can distinguish the background and target regions by the captured multi-scale features, the feature aggregation module highlights the details of different features, and the network can segment the cloud regions more accurately.

**Table 1:** Comparison of results with state-of-the-art satellite cloud image segmentation methods.

Methods	Jaccard	Precision	Recall	OA	Dice	HD	TC
Fmask	0.7519	0.7763	0.9682	0.9418	0.8245	4.381	35ms
SegCloud	0.7737	0.9038	0.8225	0.9597	0.8437	4.176	41ms
Ours	0.7912	0.9214	0.8218	0.9695	0.8692	3.935	34ms

We use VGG16, ResNet and DensetNet as the backbone network respectively, and the results are shown in Table 2, we can see that VGG16 is more suitable for our network.

**Table 2:** Comparison of results of different backbone networks.

Backbone	Jaccard	Precision	Recall	OA	Dice	HD
VGG16	0.7912	0.9214	0.8218	0.9695	0.8692	3.935
ResNet	0.7847	0.9063	0.8345	0.9566	0.8587	4.028
DenseNet	0.7896	0.9148	0.8273	0.9626	0.8604	3.992

To verify the contribution of the FFM and FCCRF modules to the network, we performed an ablation study. The results are shown in Tables 3 and 4, where we can see that the FFM module improves the performance of the network by 1.4% and the FCMCA module improves the performance of the network by 1.7%.

**Table 3:** Ablation study for FFM module.

Methods	Jaccard	Precision	Recall	OA	Dice	HD
VGG16+FAMCA+FCCRF	0.7824	0.9108	0.8336	09.581	0.8583	3.957
VGG16+FAMCA+FCCRF+FFM	0.7912	0.9214	0.8218	0.9695	0.8692	3.935

**Table 4:** Ablation study for FCCRF module.

Methods	Jaccard	Precision	Recall	OA	Dice	HD
VGG16+FAMCA+FFM	0.7819	0.9103	0.8349	0.9553	0.8549	3.962
VGG16+FAMCA+FCCRF+FFM	0.7912	0.9214	0.8218	0.9695	0.8692	3.935

## 4. Discussion

**Fig 8:**
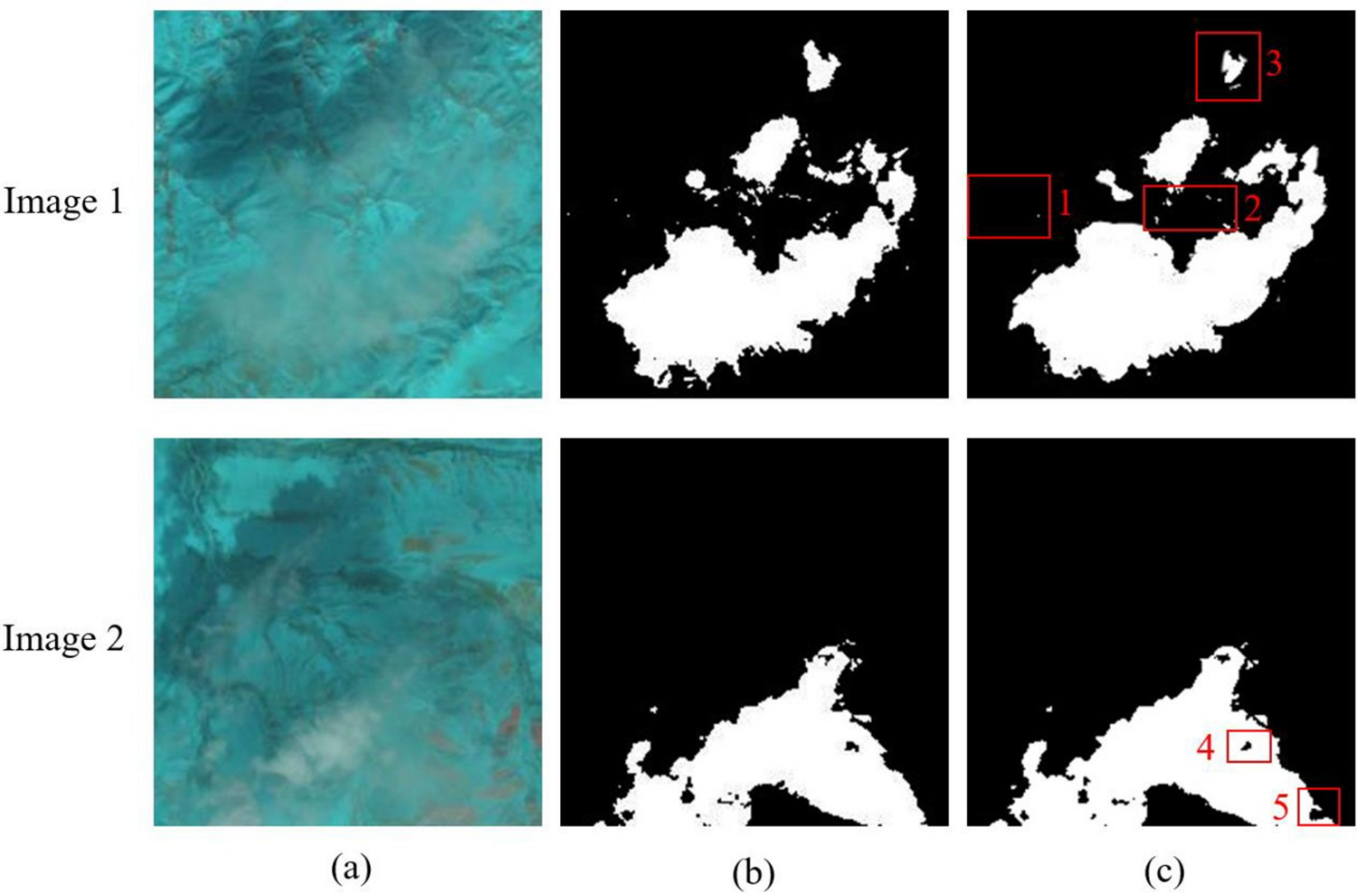
Bad samples in segmentation results, (a) are the original image, (b) are the Ground-truth, (c) are the segmentation result of the proposed method.

In the segmentation results, some samples are poorly segmented in some regions. As shown in Fig 8, in red rectangular boxes 1 and 2, isolated smaller cloud regions are incorrectly identified as ice and snow surfaces. In rectangular boxes 3 and 5, the cloud edge contours are incompletely identified. In rectangular box 4, the shape of the hole in the middle of the cloud region is not recognized accurately enough. We can further improve the accuracy of satellite cloud map segmentation by increasing the number of training data sets, improving the network structure and image contrast enhancement.

Ref [9] is sensitive to temperature and altitude, and it is easy to confuse cloud regions with ground shadows. the boundaries of cloud regions segmented by this method are blurred, and the detection accuracy is poor in mountainous areas. Ref [17] incorporates the feature maps of clouds at different levels, and the segmentation results are relatively good. However, when the ground surface is covered by ice and snow, it is easy to misidentify the ice and snow region as the cloud region. The satellite cloud image contains many small-scale targets and it has a high resolution. The low-dimensional feature map has a larger resolution, so it is more sensitive to small-scale targets and can obtain more accurate small-scale semantic information. The small number of channel attention branches and fusion module parameters ensures the segmentation speed of the network while improving the segmentation accuracy. In addition, we design a fully connected conditional random field in the output layer to solve the problem that some noise in the input image and local minima in the training process will be passed to the output layer leading to the loss of edge features, which further improves the accuracy of image segmentation.

## 5. Conclusions

The network proposed in this paper can achieve fast and high accuracy segmentation of satellite cloud images, and this network can have better performance in extracting multi-scale semantic information of satellite images, especially small-scale target semantic information. Firstly, the network uses channel attention to optimize the effective information in the feature map, which improves the extraction capability of the network for semantic information at each scale. Then, the fusion module is used to fuse the high-dimensional feature maps with the low-dimensional feature maps to enhance the network depth of the low-dimensional feature maps, which further enhances the network’s ability to obtain small-scale semantic information. With these two optimization modules, the proposed network can capture more accurate semantic information at different scales. In addition, the feature aggregation module uses an attention mechanism to automatically adjust the weights of the input multi-level features to highlight the details of different features. Finally, we design a fully connected conditional random field to address the problem that partial noise in the input image and local minima in the training process can be passed to the output layer resulting in the loss of edge features. The method proposed in this paper has a relatively simple and effective structure, which ensures its faster operation. Experimental results prove that the proposed method achieves higher segmentation accuracy with less time consumption compared to the existing advanced methods, which fully demonstrates the advancedness of this method. Proposed model improves the segmentation accuracy, but the computational consumption also increases. In the future, we can try to investigate other feature fusion methods to further reduce the complexity of the model. In addition, the number of current datasets is large, but the number of professionally labeled datasets is still small, and semi-supervised or unsupervised segmentation algorithms can be tried in the future.
